# Accumulation of neutral lipids in peripheral blood mononuclear cells as a distinctive trait of Alzheimer patients and asymptomatic subjects at risk of disease

**DOI:** 10.1186/1741-7015-7-66

**Published:** 2009-11-02

**Authors:** Alessandra Pani, Antonella Mandas, Giacomo Diaz, Claudia Abete, Pier Luigi Cocco, Fabrizio Angius, Annalisa Brundu, Nico Muçaka, Maria Elena Pais, Antonio Saba, Luigi Barberini, Cristina Zaru, Manuela Palmas, Paolo F Putzu, Alessandra Mocali, Francesco Paoletti, Paolo La Colla, Sandra Dessì

**Affiliations:** 1 Department of Biomedical Sciences and Technologies, University of Cagliari, Italy; 2 Department of Internal Medical Science, University of Cagliari, Italy; 3 Department of Cardiovascular and Neurological Science, University of Cagliari, Italy; 4 Alzheimer Center, ASL 8, Cagliari, Italy; 5 Department of Experimental Pathology and Oncology, University of Florence, Italy

## Abstract

**Background:**

Alzheimer's disease is the most common progressive neurodegenerative disease. In recent years, numerous progresses in the discovery of novel Alzheimer's disease molecular biomarkers in brain as well as in biological fluids have been made. Among them, those involving lipid metabolism are emerging as potential candidates. In particular, an accumulation of neutral lipids was recently found by us in skin fibroblasts from Alzheimer's disease patients. Therefore, with the aim to assess whether peripheral alterations in cholesterol homeostasis might be relevant in Alzheimer's disease development and progression, in the present study we analyzed lipid metabolism in plasma and peripheral blood mononuclear cells from Alzheimer's disease patients and from their first-degree relatives.

**Methods:**

Blood samples were obtained from 93 patients with probable Alzheimer's disease and from 91 of their first-degree relatives. As controls we utilized 57, cognitively normal, over-65 year-old volunteers and 113 blood donors aged 21-66 years, respectively. Data are reported as mean ± standard error. Statistical calculations were performed using the statistical analysis software Origin 8.0 version. Data analysis was done using the Student t-test and the Pearson test.

**Results:**

Data reported here show high neutral lipid levels and increased ACAT-1 protein in about 85% of peripheral blood mononuclear cells freshly isolated (*ex vivo*) from patients with probable sporadic Alzheimer's disease compared to about 7% of cognitively normal age-matched controls. A significant reduction in high density lipoprotein-cholesterol levels in plasma from Alzheimer's disease blood samples was also observed. Additionally, correlation analyses reveal a negative correlation between high density lipoprotein-cholesterol and cognitive capacity, as determined by Mini Mental State Examination, as well as between high density lipoprotein-cholesterol and neutral lipid accumulation. We observed great variability in the neutral lipid-peripheral blood mononuclear cells data and in plasma lipid analysis of the subjects enrolled as Alzheimer's disease-first-degree relatives. However, about 30% of them tend to display a peripheral metabolic cholesterol pattern similar to that exhibited by Alzheimer's disease patients.

**Conclusion:**

We suggest that neutral lipid-peripheral blood mononuclear cells and plasma high density lipoprotein-cholesterol determinations might be of interest to outline a distinctive metabolic profile applying to both Alzheimer's disease patients and asymptomatic subjects at higher risk of disease.

## Background

Alzheimer's disease (AD) is the most common progressive neurodegenerative disease affecting millions of people worldwide. The AD brain is marked by severe neurodegeneration like the loss of synapses and neurons, atrophy and depletion of neurotransmitter systems in the hippocampus and cerebral cortex [1,2]. Although we still do not know what starts the AD process, we do know that damage to the brain begins as many as 10 to 20 years before the symptoms. To date, AD diagnosis during life, and particularly of early stages of disease, is based on evaluation of multiple parameters obtained through neuropsychological testing [3], conventional imaging [4] as well as the exclusion of other neuropathologies by means of elaborate, serial and expensive procedures. Numerous progresses in the discovery, validation, and standardization of molecular biomarkers in brain or biological fluids of aid in diagnosis at different stages of AD and in the assessment of disease progression, have recently been made [5-7]. In particular, the analysis of amyloid beta-peptides (Aβ) in the liquor and the advancements in functional neuroimaging [8,9] have definitely improved the accuracy of AD diagnosis; however, it is worth saying that these procedures are available only in the best academic centres and for a limited number of patients. On the contrary, in non-specialized hospital units there are several problems to make accurate differential diagnosis of AD over a large number of subjects and also assess whether a mild cognitive impairment (MCI) might reveal early stages of disease or should rather be linked to normal aging. Therefore, a first-line test, even though not as specific as those mentioned above, yet easy to be performed and denoting systemic metabolic alterations would be an useful tool for basic and clinical AD research.

Recently, an interesting blood test for early AD diagnosis based on the expression of eighteen signaling plasma proteins was reported [10]. Waiting for the assay validation by other research groups, these findings lend further support to the hypothesis that AD patients suffer from a systemic metabolic dysfunction that beyond the brain affects also other tissues including dermal fibroblasts, which have often been employed as an in vitro model for neurological diseases, particularly AD [11,12]. Indeed, we have recently reported that skin fibroblasts from patients with diagnosis of probable sporadic AD display an imbalance between free cholesterol (FC) and cholesterol esters (CEs) pools to suggest that increased CE levels in these cells may represent an additional peripheral indicator of disease [13,14].

These metabolic alterations in dermal fibroblasts prompted us to further investigate cholesterol metabolism in freshly isolated (*ex vivo*) peripheral blood mononuclear cells (PBMCs). These cells are an ideal source for investigation due to their critical roles in immune response, metabolism, and communication with other cells and extracellular matrices almost everywhere in the human body, as well as for their ease of collection [15]. Moreover, starting from the notion that family history of AD-like dementia is considered a high-risk factor for the development of this disease [16,17] and with the aim to identify sensitive metabolic traits in support to diagnosis of probable AD, we have analyzed lipid metabolism in freshly isolated (ex vivo) PBMCs, and in plasma of AD patients and of their cognitively normal first degree relatives (AD-FDR).

As controls, we utilized cognitively normal over-65-year-old subjects (control 1) and blood donors aged 21-66 years (control 2). High levels of neutral lipids (NL), mainly CEs, in PBMCs and low high density lipoprotein-cholesterol (HDL-C) levels in plasma have been found in about 90% of patients with probable sporadic AD examined. As expected, a great variability in the NL-PBMCs data and in plasma lipid analysis of the subjects enrolled as AD-FDR was found; however, about 30% of them displayed a peripheral metabolic cholesterol pattern similar to that of the AD patient in each one of their families.

## Methods

### Participants

Blood samples were obtained from patients with possible or probable AD and from their FDR (see data reported in Table 1 and Table 2, respectively). Subjects were enrolled at the Alzheimer Center, USL 8, Cagliari, and at the Geriatric Unit of the University Hospital, Cagliari (Italy) following informed written consent from all individuals or, when necessary, from their legal guardians, under local institutional review board supervision and with approval by local Ethical Committees. Routine clinical and laboratory evaluation, including magnetic resonance imaging, was performed to exclude other causes of cognitive impairment. Patient evaluation included medical history, physical and neurological examinations, and laboratory blood tests to rule out metabolic causes of dementia (thyroid hormones, Vitamin B12, etc.), and neuroimaging (computer tomography and/or magnetic resonance) of the brain. In addition, all patients underwent neuropsychological tests, including the clinical dementia rating scale (CDR), the global deterioration scale (GDS), the Clock Drawing Test (CDT) and the Mini-Mental State Examination (MMSE) corrected for degree of schooling and age. Patients had CDR score 3-5, GDS score 6-7, CDT score < 12, and MMSE score < 24. Patients with neoplastic or hematological disorders, recent infections, severe hepatic or renal failure, myocardial infarction or cranial trauma in the previous six months, or who had received statins, antineoplastic, corticosteroid, or immunosuppressive drug treatment or surgery were not included in the present study. Additionally, 170 individuals: 57 volunteers (control 1) aged between 66-87 years, with no cognitive impairment, as established by clinical interview and by a normal CDR, GDS and MMSE score, and 113 blood donors (control 2), enrolled at the Transfusion Center, USL7, Iglesias, Italy, aged between 20-66 years (table 2), with no personal or family history of neurological or psychiatric disorders, served as controls. Given the influence of malnutrition on cholesterol metabolism, either under- or over-weight subjects have intentionally been excluded from the present study.

**Table 1:** Characteristics of control 1 subjects and Alzheimer's disease patients.

	**Control 1**			**AD**		
	
	**Total**	**Female**	**Male**	**Total**	**Female**	**Male**
Number	57	37	20	93	67	26
Age (mean ± SE)	77.2 ± 0.8	77.0 ± 1.0	77.5 ± 1.3	77.2 ± 0.7	77.9 ± 0.9	77.1 ± 1.3
t-test (C1 *vs *AD)				*P *= 0.944	*P *= 0.534	*P *= 0.206
MMSE	28.2 ± 0.2	27.9 ± 1.3	28.6 ± 0.3	18.7 ± 0.6	18.9 ± 0.6	18.3 ± 1.3
t-test (C1 *vs *AD)				*P *= 0.000	*P *= 0.000	*P *= 0.000
ORO (mean ± SE)	0.6 ± 0.1	0.6 ± 0.1	0.5 ± 0.1	2.6 ± 0.1	2.7 ± 0.1	2.5 ± 0.2
t-test (C1 *vs *AD)				*P *= 0.000	*P *= 0.000	*P *= 0.000

MMSE = Mini Mental State Examination

AD = Alzheimer's Disease

ORO = Oil Red O

**Table 2:** Characteristics of control 2 and Alzheimer's disease-First-degree relatives subjects.

	**Control 2**			**AD-FDR**		
	
	**Total**	**Female**	**Male**	**Total**	**Female**	**Male**
	
Number	113	30	83	91	66	25
Age (mean ± SE)	44.7 ± 0.9	42.2 ± 1.7	45.6 ± 1.1	46.6 ± 1.4	45.5 ± 1.4	47.3 ± 2.6
t-test (C2 *vs*. AD-FDR)				*P *= 0.229	*P *= 0.174	*P *= 0.480
ORO (mean ± SE)	0.5 ± 0.1	0.7 ± 0.1	0.4 ± 0.1	1.4 ± 0.1	1.5 ± 0.1	1.5 ± 0.2
t-test (C2 *vs*. AD-FDR)				*P *= 0.000	*P *= 0.027	*P *= 0.000

AD-FDR = Alzheimer's Disease - First Degree Relatives

ORO = Oil Red O

### Isolation of PBMCs

Blood samples were centrifuged at 2200 rpm for 15 min to separate plasma. Plasma was removed, transferred to centrifuge tubes and stored at -80°C until analysis. The buffy coat was collected and peripheral blood mononuclear cells (PBMCs) were isolated by density gradient centrifugation (Lymphoprep; density, 1.077 g/L; Nycomed Pharma, Oslo, Norway) at 1200 rpm for 10 minutes at 20°C, and washed twice with Hanks balanced salt solution (HBSS). These freshly isolated (*ex vivo*) PBMCs were utilized for the experiments. Where indicated, purified PBMCs were seeded onto 24-well plates at 1.0 × 10^4^/mL in RPMI 1640 medium with the standard supplements: (10% fetal calf serum (FCS), 200 mM L-glutamine, and 100 U/mL penicillin-streptomycin) and growth-stimulated with 10 μg/ml phytohemagglutinin (PHA; Sigma) for 24, 48 and 72 hours. The trypan blue exclusion test was used to assess cell viability.

### [^3^H]Thymidine-incorporation studies

At the time points considered, cells were incubated in triplicate for 6 h with [^3^H]thymidine (5 μCi/ml). After incubation, the cells were rinsed twice with phosphate buffered saline (PBS), and DNA was precipitated by adding 5% trichloroacetic acid at 4°C. Cells were digested with 1 M NaOH at room temperature. Aliquots were assayed for [^3^H]thymidine radioactivity and protein content.

### Cellular lipid synthesis and efflux

To determine the rate of lipid synthesis, PBMCs were incubated for 6 h with 5 μCi/ml sodium [^14^C]acetate (DuPont ± NEN; specific radioactivity 50 mCi/mmol). At 6, 18 and 36 h of incubation, cells were harvested, washed with PBS, and total lipids (TL) in cells and medium extracted with cold acetone. Lipid subclasses (FC, CE and triglycerides [TG]) were separated by thin layer chromatography (TLC TLC on kiesegel plates (Merck, Darmstadt, Germany) using a solvent system containing *n*-heptane/isopropyl ether/formic acid (60:40: 2, by vol.) and the incorporation of [^14^C]acetate into these different lipid fractions in cells and medium was measured in a Liquid Scintillation Counter. Efflux of [^14^C]TL from the cells into the medium at each time point was expressed as the percentage of the radioactivity recovered in medium/total radioactivity (cells + medium).

### Plasma lipid profile

Total cholesterol (TC) and HDL-C content was determined in plasma by using routine colorimetric enzymatic procedures (Sclavo Diagnostics International S.r.l. Sovicille, Italy).

### Neutral lipid staining

To visualize the degree of cytoplasmic neutral lipid accumulation, freshly isolated PBMCs were washed three times with PBS, and fixed by soaking in 10% formalin. Cells were then treated with isopropyl alcohol (60%), washed, and stained with oil red O (ORO) (a lipid-soluble dye which stains NL, including CE, but not FC). They appear as bright red spots in the cytoplasm, and are then counterstained with Mayer's hematoxylin. After staining, cells were imaged using a Leitz inverted-phase microscope fitted with a digital camera. At least two different fields per sample were imaged and analyzed. The red intensity was scored on a semi-quantitative scale (from 0 to 4) by two blinded observers: 0 indicated no staining; 1, rare positive cells or staining barely visible at low power (×200); 2, focal staining or faint diffuse staining clearly visible at low power; 3, multifocal staining or moderate diffuse staining; and 4, intense diffuse staining. There was significantly high correlation between the scores of the two observers (r^2 ^= 0.96; *P *= 0.000). For convenience, scores from only one observer were used for cell mean-score calculation. In some experiments ORO intensity was determined using Image J software (National Institutes of Health, United States). Red intensity was expressed as mean pixels ± SE/cm^2 ^of triplicate wells and obtained by utilizing four different selected regions of interest (ROIs).

### Statistical analysis

Data are reported as mean ± standard error (SE). Statistical calculations were performed using the statistical analysis software Origin 8.0 version (Microcal, Inc, Northampton, MA, USA). Data analysis was done using the Student t-test and the Pearson test. A value of *P *< 0.05 was considered to be statistically significant.

## Results

### Lipid profiles in freshly isolated *PBMCs from AD patients and elderly controls*

Characteristics of 93 AD patients (mean age 77.2 ± 0.7, 67 female and 26 male) and 57 controls (mean age 77.2 ± 0.8, 37 female and 20 male) enrolled for this study are given in Table 1.

**Figure 1:**
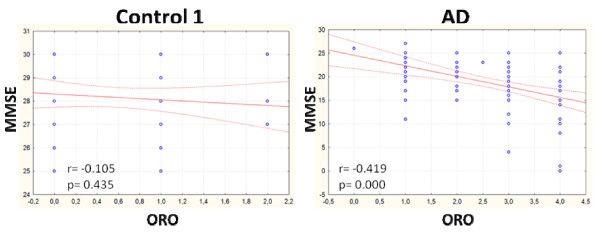
**MMSE vs ORO intensity in PBMCs from control 1 and AD**. Pearson correlation test revealed a significant inverse correlation between these variables in AD patients but not in control 1.

We initially determined the content of NL in *ex vivo *PBMCs from AD patients and age-matched controls (control 1) by staining cells with Oil Red O (ORO), a lysochrome fat-soluble dye widely used for demonstrating the presence of NL (CE and TG), which appear as bright red spots in the cytoplasm, as described above, in Materials and Methods. Cell mean ORO ± SE are reported in Table 1. As shown in Table 3, NL in control 1 PBMCs were generally absent (score 0) (49% female and 55% male) or very low (score 1) (38% female and 40% male) to indicate that intracellular lipids in the elderly are maintained at low levels, possibly by a cyclic process, as originally reported by Goldstein and Brown [18]. By contrast, NL concentration in AD cells was high, since the majority of AD-PBMCs (about 70% female and 81% male) presented ORO staining levels with a score from 2 to 4. The intensity of ORO staining in PBMCs positively and significantly correlated with the severity of the cognitive alteration, as determined by the MMSE scores in the AD population (r = -0.419; *P *= 0.000) (Figure 1). No significant correlation between MMSE and ORO staining was found in control 1 (r = 0.105; *P *= 0.435).

**Table 3:** Distribution of ORO grading in control 1 subjects and Alzheimer's disease patients.

**Score**	**Control 1**			**AD**		
	**Total**	**Female**	**Male**	**Total**	**Female**	**Male**
	
0	29	18	11	1	1	0
1	22	14	8	13	8	5
2	6	5	1	21	14	7
3	0	0	0	41	32	9
4	0	0	0	17	12	5

total	57	37	20	93	67	26

ORO = Oil Red O

AD = Alzheimer's Disease

### Lipid profiles in PHA-stimulated *PBMCs of control 1 subjects and AD patients*

In previous studies we demonstrated that, in normal PHA-stimulated PBMCs, CE synthesis increases at least 12 hours before the start of DNA synthesis, indicating that cholesterol esterification is involved in the regulation of cell-cycle progression in these cells [19-22]. In the current study we quantified DNA synthesis and NL in PHA-stimulated control 1 and AD PBMCs. DNA synthesis was measured by ^3^H-thymidine incorporation in the two cell groups cultured in parallel under identical conditions. In PHA-stimulated control 1 cells, DNA synthesis increased at 48 h of culture, reached a peak at 72 h and then declined. In AD PBMCs, increased incorporation of thymidine into DNA was already detected 24 h after PHA addition, reaching a peak at 48 h, indicating that PBMCs freshly isolated from AD patients grow at a faster rate than those from control 1 (data not shown).

**Figure 2:**
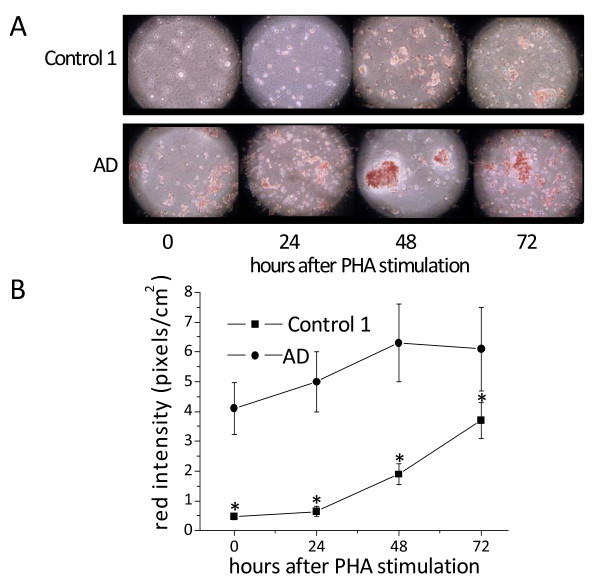
**Neutral lipid accumulation in PHA-stimulated PBMCs from control 1 subjects and AD patients**. Immediately after separation, cells were incubated with PHA for the time periods indicated. After harvesting, cells were washed, fixed by soaking in 10% formalin, stained with ORO for NL, and counter-stained with Mayer's hematoxylin for nuclei. Cells were then examined by light microscopy and two different fields per sample were imaged. Red ORO intensity was measured in these two fields using NIH Image J software. **Panel A **shows ORO staining images for one control 1 (84 years old; ORO score 0 at 0 time) and one AD (85 years old; ORO score 3 at 0 time), which are representative of about thirty subjects for each group. **Panel B **shows red intensity expressed as mean pixels ± SE/cm2, as described in Materials and Methods. *P < 0.01, compared with control 1 by Student's t test.

Changes in ^3^H-thymidine incorporation were associated with parallel changes in the content of NL. Figure 2A shows ORO images of PHA-stimulated PBMCs from one AD patient (85 years old) and one control 1 (84 years old), representing 20 AD and 20 control 1, respectively. In AD PBMCs, ORO staining was even evident at 0 h, and steadily increased at 48-72 h. Conversely, control 1 cells became ORO-positive at 48 h after PHA stimulation, and still were increasing at 72 h (Figure 2B). Also of interest is that cluster formation in AD PBMCs, indicative of cell activation, is even evident before PHA-stimulation (0 h), while it became manifest in control 1 only at 48 h after stimulation.

### Plasma lipid profile in control 1 subjects and AD patients

**Figure 3:**
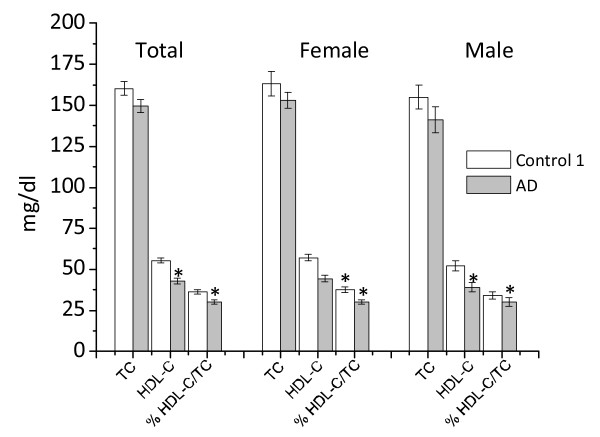
**Plasma lipid profile in control 1subjects and AD patients**. Data are expressed as a mean ± SE of the enrolled subjects. *TC *P *= 0.116; HDL *P *= 0.000; %HDL/TC *P *= 0.002; TC female *P *= 0.227; D HDL female *P *= 0.000; % HDLC/TC female *P *= 0.002; TC male *P *= 0.221; HDL male *P *= 0.003; %HDLC/TC male, *P *= 0.297 of AD vs the corresponding control 1 by Student's t test.

**Figure 4:**
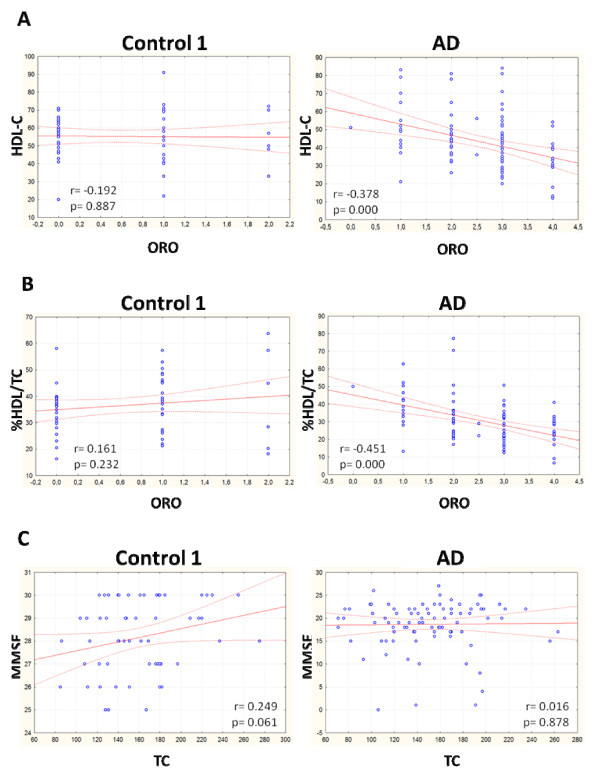
**HDL-C vs ORO intensity and MMSE vs TC in PBMCs from control 1 and AD. A. HDL-C vs ORO intensity; B. %HDL-C vs ORO intensity; C. MMSE vs TC**. The test revealed a significant inverse correlation between HDL-C and %HDL/TC vs ORO in AD patients but not in control 1. No correlation was observed between MMSE vs TC in both groups.

**Figure 5:**
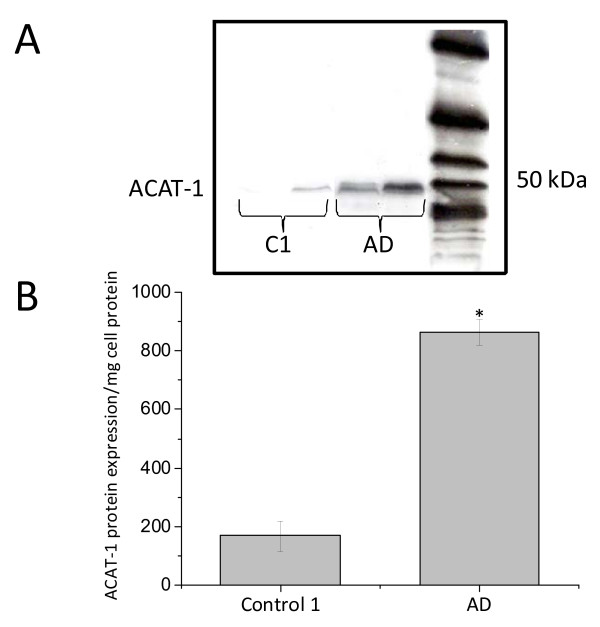
**ACAT-1 protein expression in control 1 and AD PBMCs**. Western blotting with DM10 was used to monitor ACAT-1 protein content. The anti-ACAT-1 DM10 antibody specifically recognized a single protein band from PBMC extracts with an apparent molecular weight of about 50 kDa. No other protein signal(s) was detectable. **A**. Representative ACAT-1 immunoblot. **B**. Results of densitometric analysis by Scion Image software of ACAT-1 protein immunoreactivity relative to β-actin in 20 AD patients and 20 control 1. Data values are represented as mean ± SE *P = 0.000 vs control 1.

We next measured TC and HDL-C levels in plasma collected from each blood sample after PBMCs separation. As shown in Figure 3, the levels of HDL-C in AD patients were significantly reduced, in both absolute and relative (% HDL-C/TC) terms. TC was moderately lower in AD patients as compared to subjects of control 1, but this was not statistically significant. Statistical analysis revealed that ORO staining levels negatively correlated with HDL-C levels in AD patients (r = -0.378; *P *= 0.000), but not in control 1 (Figures 4A and 4B). No significant correlation was found between plasma TC and MMSE in AD patients as well as in control 1 (Figure 4C). Additionally, Western blotting with DM10 antibody, used to monitor ACAT-1 protein, revealed that ACAT-1 protein content, in *ex vivo *PBMCs was significantly greater in AD compared to control 1 (Figures 5A and 5B).

### Cellular cholesterol metabolism and efflux

**Figure 6:**
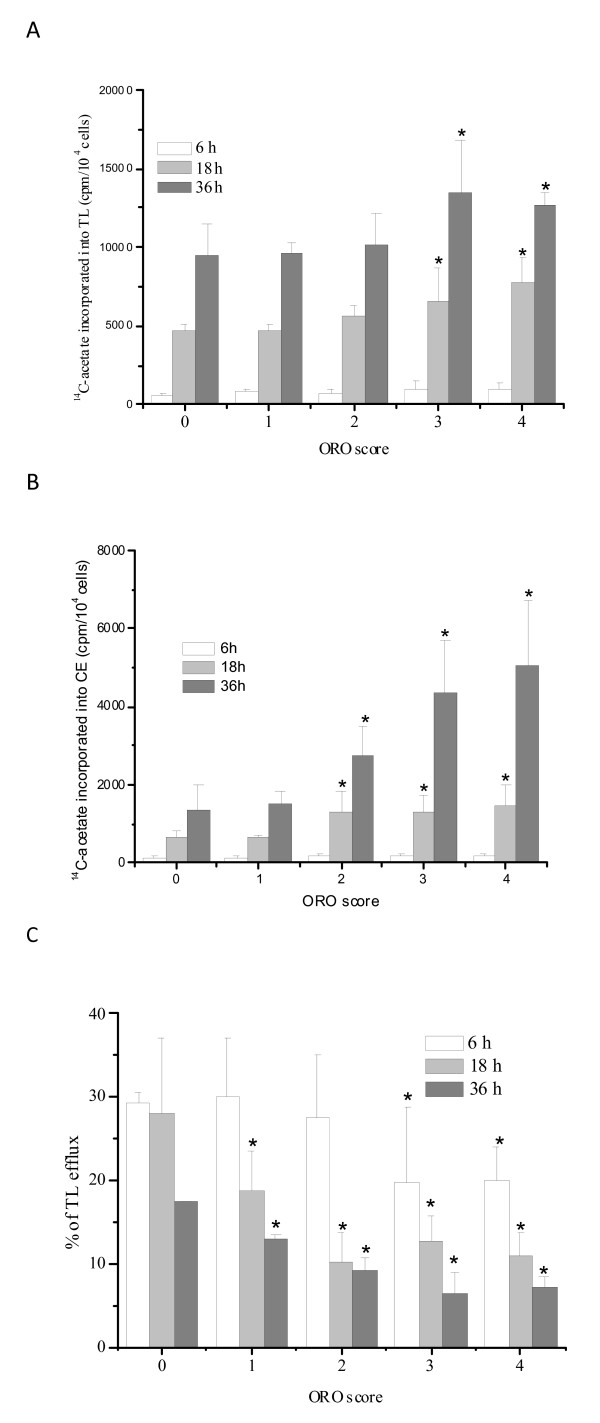
**Lipid synthesis and efflux in PBMCs with different ORO scores**. Freshly isolated PBMCs (four for each ORO score group) were plated at a density of 10,000 cell/cm^2 ^in six well plates and incubated with PHA for the time periods indicated. Total cellular lipid synthesis (A) and cholesterol ester synthesis (B) were evaluated by incubating cells for six hours in medium containing [^14^C]acetate at a final concentration of 2 μCi/ml. After incubation, cells were washed with PBS and lipids extracted with acetone. Lipid subclasses were separated out by thin layer chromatography (TLC) and [^14^C]acetate incorporation into the various lipid fractions was measured. Efflux (C) from the cells into the medium at each time point was expressed as the percentage of radioactivity in medium/total radioactivity (cells + medium). Data shown are the mean ± SE of four subjects from each score group, *P < 0.05.

Given the role of HDL in preventing cell damage by removing excess cholesterol from cells, it is conceivable that low HDL-C level in AD patients is indicative of decreased removal of lipids, mainly cholesterol, from tissues. To verify this possibility we therefore quantified FC, CE and TG syntheses, as well as lipid efflux, in PBMCs from twenty subjects, four for each ORO score group (0-4), chosen at random from the enrolled AD patients and control 1 subjects. Total lipid synthesis (TLS) and efflux values were obtained from the sum of [^14^C]acetate incorporated into FC, CE and TG fractions separated by TLC, in cells and medium. As shown in Figure 6A, TLS was slightly higher in cells with 2-4 ORO score compared to cells with 0-1 score at all time points assessed. We also noted i) a moderate but not significant rise in FC incorporation (data not shown) in cells with 2-4 ORO score, ii) a statistically significant rise in TG, and iii) highly significant increases in CE incorporation (Figure 6B). The fractional rate of lipid efflux (% of [^14^C]lipid in medium/total [^14^C]lipid) was significantly lower in 1-4 scores compared to 0 score, at all time points (Figure 6C).

### Lipid profiles in plasma and *PBMCs *of control 2 subjects and AD-FDR

It is well known that individuals who have had a late-onset AD case in their family may run an increased risk of developing AD themselves [16,17]. Therefore, we measured TC and HDL-C levels in plasma and carried out ORO staining in PBMCs from 91 FDR (mean age 46.6 ± 1.4; 66 female and 25 male) of the above AD patients (AD-FDR). We then compared data with those obtained from 113 age-matched blood donors (control 2) (mean age 44.7 ± 0.9; 30 female and 83 male) (Table 2). A high degree of variability in the ORO data and in plasma lipid analysis of the subjects enrolled as AD-FDR was observed (Table 2 and 4). However, 44% of PBMCs from AD-FDR had ORO staining levels scoring 2-3, as compared to only 9.7% of PBMCs from controls 2 (Table 4).

**Table 4:** Distribution of ORO grading in control 2 and Alzheimer's disease-First-degree relatives subjects.

**Score**	**Control 2**			**AD-FDR**		
	
	**Total**	**Female**	**Male**	**Total**	**Female**	**Male**
	
0	70	15	55	30	21	9
1	32	10	22	20	13	7
2	10	4	6	21	14	7
3	1	1	0	19	17	2
4	0	0	0	1	1	0
total	113	30	83	91	66	25

ORO = Oil Red O

AD-FDR = Alzheimer's Disease - First Degree Relatives

**Figure 7:**
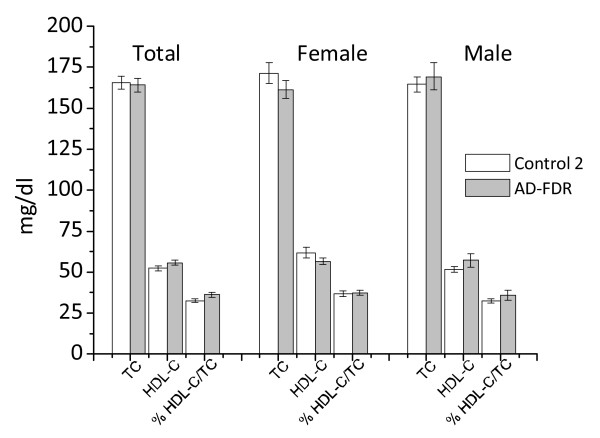
**Plasma lipid profile in control 2 and AD-FDR**. Data are expressed as a mean ± SE of the enrolled subjects. *TC *P *= 0.784; HDL *P *= 0.154; %HDL/TC *P *= 0.04; TC female *P *= 0.185; D HDL female *P *= 0.304; % HDLC/TC female *P *= 0.05; TC male *P *= 0.490; HDL male *P *= 0.215; %HDLC/TC male, *P *= 0.316 of AD-FDR vs the corresponding control 2 by Student's t test.

**Figure 8:**
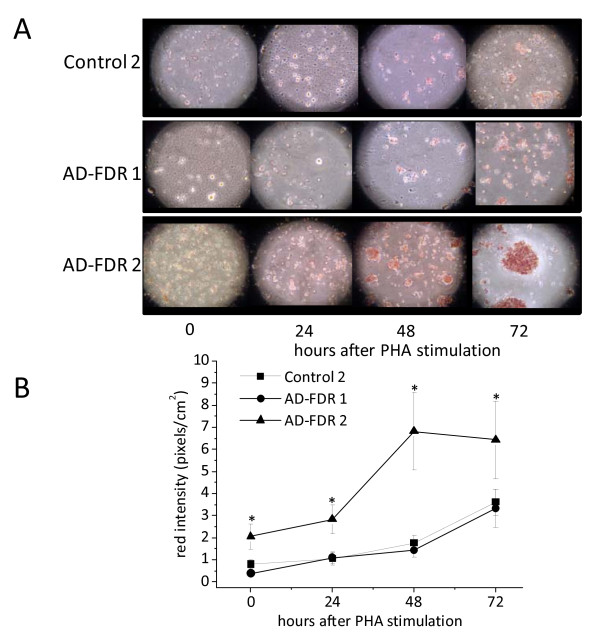
**Neutral lipid accumulation in PHA-stimulated PBMCs from control 2 and AD-FDR subjects**. Immediately after separation cells were incubated with PHA for the indicated time periods. After harvesting, cells were washed, fixed by soaking in 10% formalin, stained with ORO for NL, and counter-stained with Mayer's hematoxylin for nuclei. Cells were then examined by light microscopy and two different fields per sample were imaged. Red ORO intensity was measured in these two fields by using NIH Image J software. **Panel A **shows images of ORO staining of one control 2 (47 years old) and two AD-FDR one (48 years old) with ORO score 0 and the other one (37 years old) with ORO score 3 at 0 time. These images are representative of at least 30 control 2, 15 AD-FDR with ORO score between 0 and 1, and 15 with ORO score between 2 and 3. **Panel B **shows red intensity expressed as mean pixels ± SE/cm^2 ^of triplicate wells obtained by utilizing four created different regions of interest (ROIs). *P < 0.01, compared with control 2 by Student's t test.

**Figure 9:**
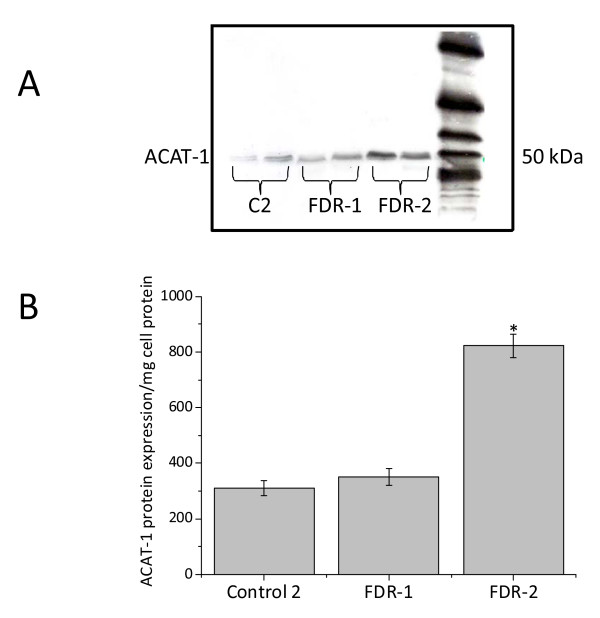
**ACAT-1 protein expression in PBMCs from control 2 and AD-FDR subjects**. Western blotting with DM10 was used to monitor the ACAT-1 protein content. The anti-ACAT-1 DM10 antibody specifically recognized a single protein band from cell extracts of PBMCs, with an apparent molecular weight of 50 kDa. No other protein signal(s) was detectable. **A**. Representative immunoblot of ACAT-1 of two control 2; two AD-FDR with ORO score between 0 and 1 (FDR-1) and two AD-FDR with ORO score between 2-3 (FDR-2). **B**. Results of densitometric analysis by Scion Image software of ACAT-1 protein.

TC and HDL-C levels did not significantly change in FDR plasma (Figure 7). However, HDL-C levels inversely correlate with ORO scores, even though not significantly (data not shown). Interestingly, three unaffected relatives of comparable age (two brothers and one sister) of three AD patients did not have relevant accumulation of neutral lipids in their PBMCs, nor had lower levels of HDL-C (data not shown). When PBMCs were stimulated with PHA, about half the PBMCs exhibited ORO kinetics similar to those of AD patients, while the other half were similar to controls 2 (Figures 8A and 8B). In agreement with these data, ACAT-1 Western blotting analysis revealed that about 50% of *ex vivo *FDR PBMCs show a significant increase in ACAT-1 protein levels compared to controls 2 (Figures 9A and 9B).

## Discussion

Cholesterol is essential for the organization of liquid-ordered microdomains (rafts) in the plasma membrane, which serve as a scaffold for the proper assembling and functioning of a variety of membrane-associated proteins, including β- and γ-secretases [23,24] that are involved in the Aβ formation. Progressive accumulation of Aβ in the brain is considered a pathogenetic event common to all forms of AD [25]. This suggests that alterations in cholesterol homeostasis and, more specifically, in raft organization may also have a great influence on AD development and progression [26-28]. In particular, CEs, rather than FC, seem to be directly correlated with Aβ production. It has, in fact, been reported that Aβ generation is modulated by the activity of acyl-coenzyme A: cholesterol acyltransferase (ACAT), the enzyme that catalyses the formation of CEs inside cells [29,30]. Inhibitors of ACAT were reported to strongly reduce Aβ production in cell and AD animal-based models [31,32]. In depth, it was reported that in a mouse model of AD the ACAT inhibitor CP-113,818 reduced by 90% amyloid pathology as compared to transgenic mice which received placebo [32]. Findings reported here show significantly higher neutral lipid levels (mainly CE) and increased ACAT-1 protein amounts in at least 85% of PBMCs freshly isolated (*ex vivo*) from patients with probable sporadic AD as compared to cognitively normal age-matched controls. These data are in keeping with and extend our preliminary findings showing higher NL levels in AD PBMCs [33], and further support the hypothesis that intracellular CE levels are systemically increased in AD-patients. That said, at least in theory, one might expect that cholesterol levels in the plasma HDL fraction, which is responsible for removing the excess of cholesterol from cells, will undergo a decrease [34]. In accordance with this, a recent study of middle-aged adults reports a link between low HDL-C and memory loss. Decreasing HDL-C was associated with memory decline over a five-year follow-up period; while no link was found between TC and TG plasma concentrations and memory deficit or decline [35]. Similarly, we found a significant reduction, both in absolute and relative terms, in HDL-C levels in plasma from AD blood samples as compared to age-matched controls. A moderate reduction in TC was also observed by us in AD patients. Additionally, correlation analyses revealed a positive correlation between HDL-C and MMSE, as well as between HDL-C and CE accumulation, as determined by ORO staining in AD patients. Although the significance, if any, of the observed correlation between low HDL-C levels and AD risk remains to be established, our data indicate that the low HDL-C levels in the plasma of AD patients may be a consequence of a decreased removal rate of cholesterol from tissues, as a result of the increased entrapment of CEs by cells. In reality, the fractional rate of lipid efflux (% of ^14^C-lipid in medium/total^14^C-lipid) was significantly lower in those cells with high ORO scores compared to those with low scores. It therefore seems that low HDL-C levels, coupled with lipid accumulation in peripheral cells, represent an additional index of AD. Interestingly, no correlation was found between total plasma cholesterol and MMSE in AD patients, indicating that, contrary to what has been reported in several other studies, hypercholesterolemia *per se *does not appear to be a risk factor for AD [36-38].

However, although it is generally accepted that alterations in cholesterol metabolism are a critical event in AD, it is still unclear whether they are the cause or the effect of disease and, actually, we cannot completely exclude the possibility that changes in lipid metabolism may be due to factors other than AD. Dementia occurs late in life, but it is increasingly recognized that there is a long preclinical phase characterized by progressive neuropathological changes that become clinically detectable with age. The "life-long" view of dementia contributes to stress the importance of risk factors in midlife [39]. Individuals who have had a late-onset AD case in their family may run an increased risk of developing AD themselves [17,18], thus the analysis of lipid metabolism in these subjects could be a suitable biochemical approach to monitor changes in cholesterol homeostasis before any cognitive decline might occur. We observed great variability in the ORO data and in plasma lipid analysis of the subjects enrolled as AD-FDR. However, 44% of PBMCs from AD-FDR had ORO staining levels that scored from 2 to 3, as compared to only 9.7% of PBMCs from controls.

## Conclusion

Overall, the assessment of cholesterol ester metabolism in PBMCs ex vivo by means of a reliable assay that can be easily and quickly performed might be a very useful biochemical tool in support to AD diagnosis. In addition, the fact that some AD-FDR tend to display a metabolic cholesterol pattern similar to that of AD patients, may suggest that the determination of PBMC ORO staining and plasma HDL-C levels may be useful for identification of a subset of midlife subjects who might run a major risk of developing AD; and the identification of incipient AD-related cognitive impairment would be the basis for initiation of treatment with drugs that might slow or stop the degenerative process, such as ACAT inhibitors [29-31]. Above all it remains to be assessed whether: i) changes in NL are also present in PBMCs from patients with non-AD neuropathologies such as for instance vascular, Lewy body and frontotemporal dementias; ii) asymptomatic individuals with decreased plasma HDL-C and increased PBMC lipids will really go on towards Alzheimer's disease over time.

## Abbreviations

ACAT: Acyl-coenzyme A: Cholesterol AcylTransferase; AD: Alzheimer's Disease; AD-FDR: Alzheimer's Disease - First Degree Relatives; Aβ: Amyloid Beta-peptides; CDR: Clinical Dementia Rating scale; CDT: Clock Drawing Test; CEs: Cholesterol Esters; FC: Free Cholesterol; FCS: Fetal Calf Serum; FDR: First Degree Relatives; GDS: Global Deterioration Scale; HBSS: Hanks Balanced Salt Solution; HDL-C: High Density Lipoprotein - Cholesterol; MCI: Mild Cognitive Impairment; MMSE: Mini Mental State Examination; NL: Neutral Lipid; NL-PBMCs: Neutral Lipid - Peripheral Blood Mononuclear Cells; ORO: Oil Red O; PBMCs: Peripheral Blood Mononuclear Cells; PBS: Phosphate Buffered Saline; PHA: PhytoHemaAgglutinin; ROIs: Regions Of Interest; SE: Standard Error; TG: Triglycerides; TL: Total Lipid; TLC: Thin Layer Chromatography; TLS: Total Lipid Synthesis.

## Competing interests

This study was supported by grants from Fondazione Regione Autonoma della Sardegna and Fondazione Monte dei Paschi di Siena. The authors state that there are no conflicts of interest that may have influenced this work and that some findings in this study have been the subject of patent application no.PCT/IT2007/000110 "Methods for the diagnosis of proliferative and/or conformational diseases" (S. Dessì, P. La Colla, and A. Pani).

## Authors' contributions

AP, AM and SD drafted the manuscript, participated in the design and coordination of the study. CA, PLC, FA, AB, NM, MEP, AS, CZ and MP performed experimental procedures and clinical briefcases of AD patients, and collected data regarding FDR and controls. GD and LB performed the statistical analysis. PFP, PLC, FP and AM commented on the manuscript and participated in the design. All authors read and approved the final manuscript.

## Pre-publication history

The pre-publication history for this paper can be accessed here:


